# Gender effects on autism spectrum disorder: a multi-site resting-state functional magnetic resonance imaging study of transcriptome-neuroimaging

**DOI:** 10.3389/fnins.2023.1203690

**Published:** 2023-06-20

**Authors:** Yanling Li, Rui Li, Ning Wang, Jiahe Gu, Jingjing Gao

**Affiliations:** ^1^School of Electrical Engineering and Electronic Information, Xihua University, Chengdu, China; ^2^School of Information and Communication Engineering, University of Electronic Science and Technology of China, Chengdu, China

**Keywords:** autism spectrum disorder, gender effect, resting-state fMRI, neurotranscriptome, default mode network, multisite

## Abstract

**Introduction:**

The gender disparity in autism spectrum disorder (ASD) has been one of the salient features of condition. However, its relationship between the pathogenesis and genetic transcription in patients of different genders has yet to reach a reliable conclusion.

**Methods:**

To address this gap, this study aimed to establish a reliable potential neuro-marker in gender-specific patients, by employing multi-site functional magnetic resonance imaging (fMRI) data, and to further investigate the role of genetic transcription molecules in neurogenetic abnormalities and gender differences in autism at the neuro-transcriptional level. To this end, age was firstly used as a regression covariate, followed by the use of ComBat to remove the site effect from the fMRI data, and abnormal functional activity was subsequently identified. The resulting abnormal functional activity was then correlated by genetic transcription to explore underlying molecular functions and cellular molecular mechanisms.

**Results:**

Abnormal brain functional activities were identified in autism patients of different genders, mainly located in the default model network (DMN) and precuneus-cingulate gyrus-frontal lobe. The correlation analysis of neuroimaging and genetic transcription further found that heterogeneous brain regions were highly correlated with genes involved in signal transmission between neurons’ plasma membranes. Additionally, we further identified different weighted gene expression patterns and specific expression tissues of risk genes in ASD of different genders.

**Discussion:**

Thus, this work not only identified the mechanism of abnormal brain functional activities caused by gender differences in ASD, but also explored the genetic and molecular characteristics caused by these related changes. Moreover, we further analyzed the genetic basis of sex differences in ASD from a neuro-transcriptional perspective.

## Introduction

The gender differences have been considered to be one of the causes of physiological heterogeneity of neurological disorders. Autism Spectrum Disorder (ASD) is a highly heritable psychiatric disorder with significant gender differences (Rossignol and Frye, 2012). Its main characteristics include a lack of social communication skills, restricted or repetitive behaviors or interests (Matta et al., 2019). Uncertainty persists regarding the connection between neurological impairment and the genetic transcription of ASD. Male have a higher susceptibility to ASD than female. To be more precise, the male-to-female ratio is close to 4:1 (Loomes et al., 2017; Maenner et al., 2020). However, this quantitative bias toward male in existing studies has results in ignoring the influence of gender bias on pathophysiology and genetic mechanisms, thereby impeding the generalizability of research findings (Werling and Geschwind, 2013).

Functional Magnetic resonance imaging (fMRI) provides an effective tool for the identification and investigation of altered structural and functional brain networks. Researchers can precisely identify heterogeneous brain areas using MRI with high pixel resolution. The failure of the default mode network (DMN) is involved in a variety of psychiatric diseases. The local functional changes of DMN and their atypical developmental trajectory are considered to be the typical neurobiological features of ASD (Padmanabhan et al., 2017). In a longitudinal study of gray matter in the brains of female with ASD, abnormal growth features were found to be more pronounced in females than in males, male patients showed increased activation in the bilateral medial frontal gyrus and female patients showed decreased activation in the midbrain and limbic regions (Schumann et al., 2010; Schneider et al., 2013). In contrast, the volume of gray matter, white matter, and hippocampus was larger in male ASD than in HC, whereas females did not experience these changes. In addition, the volume of the right putamen was smaller in females with ASD than in HC, but no such difference was found in males (Zhang et al., 2018). Although this sex difference is not unique to autism, the study of sex differences is crucial in revealing the underlying pathogenesis of ASD.

With the development of genetic transcription technology, more and more targeted researchers have found that specific gene expression patterns may play an important role in the pathogenesis of ASD (Quesnel-Vallieres et al., 2019). It suggests that transcriptomics may also influence the sex aberration in ASD. However, to date, research on sex-specific susceptibility genetic risk loci for autism is incomplete (Modabbernia et al., 2017). Studies have reported that there appears to be a “female protective pattern” in women with a higher threshold of genetic susceptibility (Robinson et al., 2013; Werling and Geschwind, 2013; Jacquemont et al., 2014). In addition, another study points out that fetal testosterone levels in amniotic fluid were positively correlated with typical behavior after maturation, while women experience chronic exposure to higher levels of androgens before birth and these high levels of androgens seem to shelter them from ASD (Auyeung et al., 2009).

In this study, we aimed to use resting-state functional magnetic resonance imaging to calculate the functional activity intensity of the Blood Oxygen Level Dependent signal (BOLD) in Amplitude of Low Frequency Fluctuation (ALFF), Degree Centrality (DC), and Regional Homogeneity (ReHo; Zang et al., 2004; Zuo et al., 2012; Yan et al., 2013; Lin et al., 2023; Wang et al., 2023) to describe the neural activity of male and female with ASD. We then use ComBat (Fortin et al., 2018; Reardon et al., 2021; Chen et al., 2022; Du et al., 2023) to remove the “scanner effect” and obtain cross-site large sample heterogeneous brain functional activity changes. Finally, we use partial least squares regression analysis (PLSr) to characterize the spatial correlation between changes in brain functional activity characteristics and genetic transcription profiles of ASD.

## Materials and methods

### Participant

To investigate the effects of gender differences on the brains of individuals with autism, we utilized rigorous criteria to select resting state fMRI data from autism brain imaging data exchange (ABIDE)^1^ of 139 male ASD, 153 male healthy control (HC), 34 female ASD, and 46 female HC, totaling 372 subjects (Di Martino et al., 2014). In order to reduce individual differences and multi-site data differences, we applied the following data screening criteria: (1) Head motion displacement was less than 2 mm or 2 degrees. (2) The average frame displacement is less than 0.5. (3) The missing volume ratio is less than 0.5. (4) The Chi-square test *value of p* of male and female subjects at each site was greater than 0.05.

### Preprocessing of resting state fMRI data

All resting state fMRI data were preprocessed by DPARSFA in a standardized and unified channel, which included the following 9 steps: (1) The first 10 volumes were deleted. (2) The remaining images were aligned, and the data with maximum head movement exceeding 2 degrees was removed. (3) All fMRI data were matched to the standard Echo Planar Imaging (EPI) template from the Montreal Neurological Institute (MNI-152) and resampling to a 3×3×3 mm voxel size. (4) The linear trend of fMRI data was removed. (5) The ALFF map of each subject was calculated and normalized using Z-transformation. (6) A bandpass filter was used to remove the noise signal (0.01 < *f* < 0.1 Hz) for the remaining BOLD time series. (7) Nuisance covariates from white matter and Cerebro-Spinal Fluid (CSF) were estimated separately and were then regressed from the resting-state fMRI data along with the motion realignment parameters, global mean signal. (8) To further reduce the effect of head movement, cubic spline interpolation was used to scrub the volume of the image with an average Frame Displacement (FD) > 0.5 mm. (9) The ReHo and DC images of each subject were normalized by z transformation. ALFF, ReHo, and DC use 8 mm Full Width at Half Maxima (FWHM) Gaussian kernel for spatial smoothing.

### Measures of brain functional activity

In this study, to quantify the characteristics of brain functional activity, functional activity intensity, local and global functional connectivity were utilized to characterize the abnormal brain functional activity of autistic patients. In particular, ALFF was mainly used to reveal the intensity of the low-frequency BOLD signal in the region of spontaneous activity frequency between 0.01 and 0.1, which is generally considered to be a neural signal. The local functional connectivity model is characterized by ReHo to describe the trend of consistency between the time series of a given voxel and the time series of its 26 nearest voxels. Additionally, the global functional connectivity model was measured by DC, which describes the correlation coefficients between a given voxel time series and the whole brain voxel time series, and calculated as the mean of the correlation coefficients between all other voxels with a correlation coefficient greater than 0.25.

### Intergroup analysis

To eliminate the “scanner effect” caused by the use of different MRI scanners and acquisition protocols at data acquisition sites, the variation which may affect the characterization of imaging features and lead to spurious findings need to be addressed. To prevent the interference of such unnecessary sources of variation in the experiment, we utilized ComBat to remove site effects while using age as a covariable. After controlling for age differences, the two-sample *t*-test was used to determine the whole-brain differences in ALFF, DC, and ReHo between ASD and HC. Statistical *t*-map were obtained, with a minimum cluster size >20.

### Harmonization decoding of functional cognitive patterns

After identifying heterogeneous brain regions in ALFF, ReHo, and DC associated with autism, we used the Neurosynth online analysis tool^2^ to analyze the cognitive functions most relevant to these heterogeneous brain regions. Specifically, we divided FDR-corrected *t*-maps into two groups: those showing increased functional activity (positive) and those showing decreased functional activity (negative), which were input into Neurosynth. Finally, the top 10 behavioral cognition terms with the greatest correlation were selected as typical representatives of regional functions.

### Prediction of clinical evaluation using brain functional activity

To evaluated whether functional activity in heterogeneous brain regions predicts clinical social performance in patients with ASD, we utilized the functional activity characteristics of these heterogeneous brain regions, as well as the data from Autism Diagnostic Interview-Revised (ADI-R) and Autism Diagnostic Observation Scale (ADOS) as input for the support vector regression model. Age is taken as the covariable of the training model, and our estimated probability is verified for accuracy by the leave-one out cross-validation method. Finally, the SVC training model’s performance was assessed using the 10-fold cross-validation approach, which employed the functional activity values of the identified heterogeneous brain areas as the feature input.

### Matching of brain regions to gene expression estimates

In this study, we utilized the whole human brain gene expression dataset provided by the Allen Human Brain Atlas (AHBA),^3^ which collected 3,702 different parts of brain tissue (Age = 42.50 ± 13.38 years old; Male/female = 5/1) from six healthy individuals post-mortem. Abagen was used to re-annotate gene samples and match gene expression with the brain region of Brodmann Atlas.^4^ The following steps were taken: (1) the microarray expression matrix was re-annotated and gene probes of invalid Entrez-ID were removed; (2) the probes with expression intensity lower than the background value were discarded if they were present in less than 50% of the donor samples. After filtration, a total of 31,569 gene probes were obtained; (3) the probe whose regional expression pattern is most consistent with that of RNA-seq is selected and used in case multiple gene probes correspond to the same gene. This is determined by calculating the Spearman rank correlation between the microarray expression of each probe and the RNA-seq expression data of the corresponding gene is calculated, and selecting the probe with the highest correspondence.

### Associations between functional differences and gene expression profiles

Based on the correlation analysis between the functional activities of heterogeneous brain regions and gene transcription, PLS regression^5^ was utilized to identify different expression patterns of weighted linear combinations of all genes. Specifically, in the experiment, gene expression transcription profiles of specific brain regions were used as predictive variables, while functional activity characteristics of specific brain regions were used as response variables. To correct for spatial autocorrelation of the functional feature data, we used permutation analysis to reorder the functional feature data (*n* = 10,000) to test the statistical significance of the maximum explainable variance information of the original arrangement in each PLS dimension (Li et al., 2021). Subsequently, to verify the spatial similarity between the weighted gene expression pattern obtained by PLS and brain functional characteristics, displacement analysis (*n* = 10,000) was performed to reorder the weighted gene expression pattern.^6^ Finally, Bootstrapping was used to perform random sampling on the gene weights of the significant dimensions of PLS results to estimate the gene variability, calculate the ratio of PLS gene weights to the standard error of gene variability, and then sequence the final scores to get the genes significantly related to brain functional activities.

In addition, we matched significant gene weights obtained through PLS to brain regions to obtain weighted gene expression maps.

### Expression analysis of autism-related genes

To further analyze the heterogeneous expression of genes between males and females, we collected 90 identified ASD-related genes from AHBA^7^ and downloaded whole-brain expression maps of these genes from Neurosynth.^8^ The gene expression map was then divided into 82 subregions using the Brodmann template, and the average expression levels of 90 risk genes in each subregion were calculated and standardized. The co-expression patterns between genes were analyzed using Pearson correlation. To further verify the significance of co-expressed gene expression patterns, we further analyzed the spatial expression patterns of 1,028 ASD-related genes identified in SFARI.^9^ In addition, gene temporal-specific expression analysis was used to characterize the expression intensity locus of genes of interest collected from 50 to 30,000 days in 11 neocortical areas.^10^

### Gene enrichment analysis

To further explore the biological function of the specifically expressed genes and their unique genetic pathways, we employed Metaspace analysis^11^ for gene enrichment analysis. The top 5% of the genes with the weight value after PLS sequencing were included in the gene enrichment analysis. The significance threshold of gene pathway and process enrichment analysis was 0.05, and the results were significantly corrected by the FDR-BH method. Tissue-specific expression analysis tools (TSEA)^12^ were used to further determine whether ASD-related genes found by PLS were specifically expressed in different human tissues. A specific expression index (PSI = 0.05, 0.01, 0.001, and 0.0001) was used to determine the significance level of tissue-specific expression of the gene.

## Results

### Demographic characteristics

A total of 461 subjects ranging 5 from 56 years of age were enrolled from six independent sites in this study, namely PITT, YALE, NYU I, CALTECH, SDSU, and EMC. Supplementary material details the specifics and scanning parameters of each site. Notably, there was no significant difference in age between the ASD and HC groups at each site (for details, please refer to Table 1).

**Table 1:** The demographic information of the used subjects.

Male (ASD = 139, HC = 153)								
Site	Groups	N	Age	*p* value	ADOS: total	ADOS: severity	ADOS: RRB	SRS: motivation
Pitt	ASD	16	21.4 ± 7.88	0.51	11.4 ± 3.5	/	/	/
	HC	18	19.7 ± 6.88		/	/	/	/
Yale	ASD	16	13.1 ± 3.33	0.53	12.1 ± 3.2	7.2 ± 1.6	2.5 ± 1.3	/
	HC	17	12.4 ± 3.01		/	/	/	/
SDSU	ASD	21	13.4 ± 2.93	0.89	14.7 ± 4.6	7.9 ± 1.9	3.6 ± 1.7	78.0 ± 10.4
	HC	19	13.6 ± 3.35		/	/	/	45.9 ± 5.8
NYU_I	ASD	62	14.5 ± 6.55	0.19	11.5 ± 4.2	7.2 ± 2.0	3.4 ± 1.5	/
	HC	73	16.0 ± 6.42		/	/	/	/
EMC	ASD	12	8.75 ± 1.26	0.11	/	/	/	/
	HC	12	8.02 ± 0.88		/	/	/	/
Caltech	ASD	12	25.5 ± 6.68	0.14	13.0 ± 4.4	/	/	/
	TD	14	31.5 ± 11.8		/	/	/	/

Female (ASD = 34, HC = 46)								
Site	Groups	N	Age	*p* value	ADOS: total	ADOS: comm	ADOS: social	SRS: total
Pitt	ASD	3	13.0 ± 0.64	0.21	12.1 ± 3.6	4.3 ± 0.95	7.9 ± 3.0	/
	HC	4	16.1 ± 3.60		/	/	/	/
Yale	ASD	7	13.5 ± 2.64	0.79	12.5 ± 3.5	4.6 ± 1.3	7.9 ± 2.5	90.5 ± 40.4
	HC	7	13.9 ± 2.63		/	/	/	17.7 ± 13.7
Caltech	ASD	4	24.4 ± 6.91	0.71	13.6 ± 4.9	4.8 ± 1.6	8.8 ± 3.4	/
	HC	4	22.8 ± 4.31					/
NYU_I	ASD	10	18.9 ± 8.82	0.19	11.2 ± 3.9	3.7 ± 1.4	7.4 ± 3.1	93.8 ± 33.1
	HC	26	15.4 ± 6.45			/		
SDSU	ASD	7	13.9 ± 3.20	0.37	13.7 ± 2.9	/	/	93.1 ± 13.0
	TD	2	11.6 ± 0.99		/	/	/	/
EMC	ASD	3	7.92 ± 0.94	0.90	/	/	/	/
	TD	3	7.82 ± 0.93		/	/	/	/

### Heterogeneous brain regions associated with ASD

**Figure 1:**
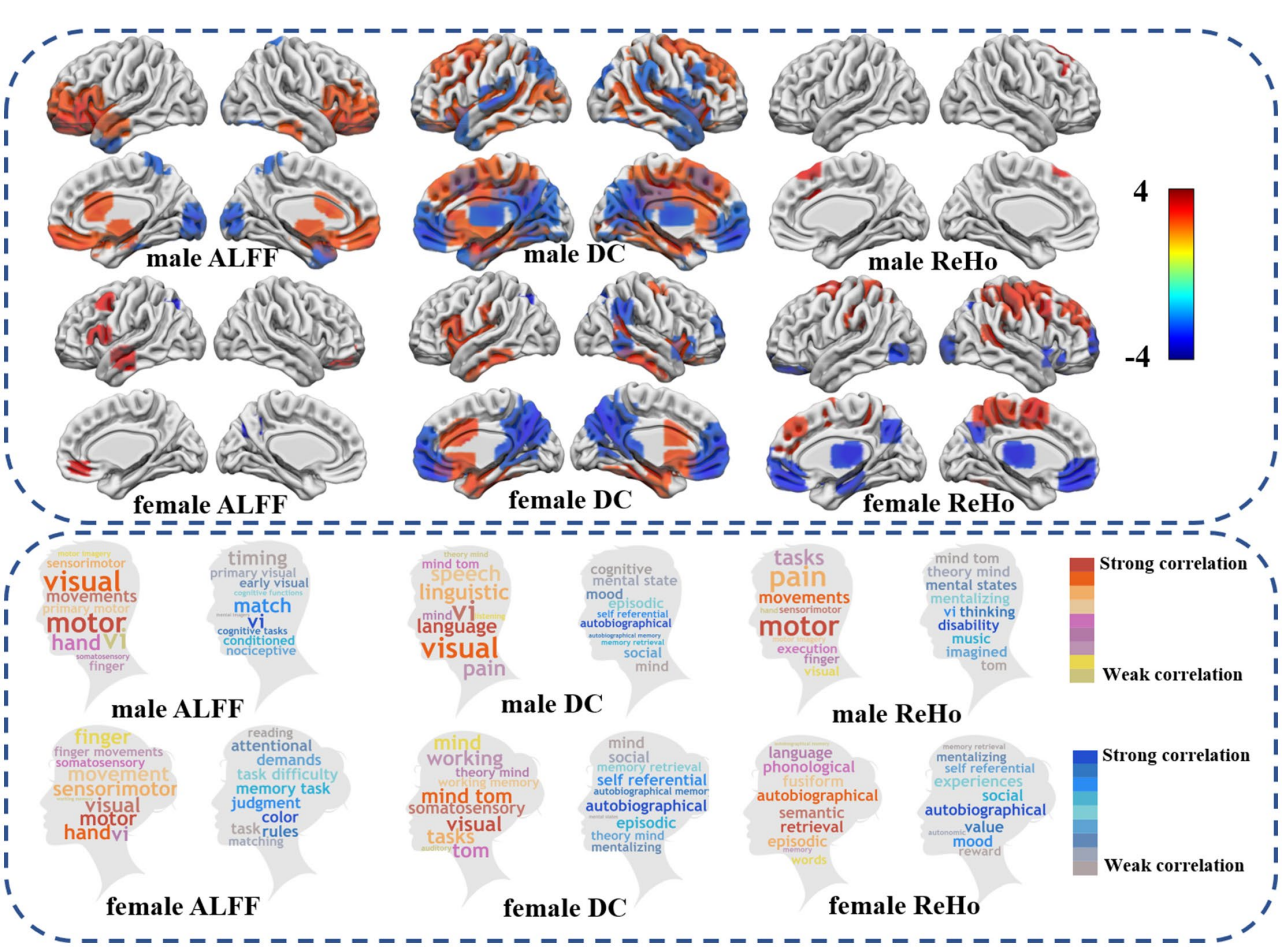
Differences in fMRI between autism spectrum disorders and healthy controls. The upper half showed differences between ASDs and HC in low frequency brain functional activity, global functional connectivity, and local functional connectivity similarity (FDR *p* < 0.05). The lower part of the word cloud map shows the difference between ASD and HC and the brain regions involved in the cognitive function of the brain. Warm colors show an increase in the functional activity of ASD compared with HC, while cool colors show a decrease in the functional activity of ASD compared with HC.

The neuroimaging analysis of this study revealed distinct alterations in brain functional activity, similarity of local functional activity, and global functional activity among females and males with autism. The group differences are shown in Table 2; Figure 1.

**Table 2:** Significant group differences in amplitude of low-frequency fluctuation (ALFF), local regional homogeneity (ReHo), and degree centrality (DC) between children with ASD and HC, and between adults with ASD and HC.

Brain region	MNI coordinates			Cluster size (voxels)	*T*-value
	*x*	*y*	*z*		
Female increase					
DC					
Superior frontal gyrus, medial	−3	63	12	239	4.7604
Inferior frontal gyrus, orbital part	51	18	−9	23	3.9837
Precuneus	0	−78	48	840	5.5405
Supramarginal gyrus	57	−48	36	46	4.1873
ReHo					
Orbitofrontal area	−9	39	−27	148	5.3124
Parahippocampal gyrus	24	−18	−18	30	4.9182
Thalamus	3	−21	12	32	4.7798
Precuneus	3	−66	36	32	4.6103
Orbital part of left inferior frontal gyrus	−18	21	−24	87	4.92
Orbital part of right superior frontal gyrus	15	21	−24	62	4.31
Female decrease					
ALFF					
Middle temporal gyrus	−60	−12	−12	24	−4.9722
Inferior frontal gyrus	−60	18	12	23	−5.1833
Middle frontal gyrus	−39	12	51	26	−5.2835
DC					
Caudate nucleus	15	18	−6	242	−4.7347
Median cingulate and paracingulate gyri	9	9	33	26	−4.4328
Postcentral gyrus	−51	−21	30	22	−4.427
ReHo					
Precental gyrus	42	6	42	29	−4.5935
Postcentral gyrus	27	−42	48	25	−4.3321
Supplementary motor area	−12	0	60	97	−6.0051
Superior frontal gyrus, dorsolateral	21	−3	54	47	−4.3424
Male increase					
ALFF					
Temporal pole: middle temporal gyrus	−27	12	−42	49	3.8309
Calcarine fissure and surrounding cortex	0	−93	6	99	4.0417
DC					
Temporal pole: middle temporal gyrus	33	18	−42	29	3.4392
Rolandic operculum	57	−12	9	244	4.5682
Temporal pole: superior temporal gyrus	−48	21	−21	51	4.3799
Middle frontal gyrus, orbital part	0	57	−6	351	4.8667
Posterior cingulate gyrus	0	−51	21	709	4.6348
Superior temporal gyrus	−66	−18	9	119	5.1184
Superior occipital gyrus	21	−87	36	48	3.7566
Precental gyrus	48	9	33	29	3.7216
ReHo					
Thalamus	−3	−18	12	31	4.6367
Posterior cingulate gyrus	0	−42	30	141	4.6859
Supramarginal gyrus	63	−27	30	39	4.2962
Male decrease					
ALFF					
orbitofrontal cortex	−21	21	−18	1973	−5.4675
Orbital part of inferior frontal gyrus	18	30	−27	44	−3.6374
Middle temporal gyrus	−60	3	−18	30	−3.6395
Middle temporal gyrus	57	0	−15	20	−3.4845
Insula	36	12	−12	51	−4.6197
Putamen	27	−15	−6	43	−4.1296
Inferior frontal gyrus, opercular part	48	15	12	25	−3.5511
Middle frontal gyrus	33	36	18	26	−3.8883
Supramarginal gyrus	45	−42	36	24	−3.5924
DC					
Superior frontal gyrus, dorsolateral	24	15	39	1,420	−5.7972
Middle temporal gyrus	−42	−48	9	843	−5.0984
Middle frontal gyrus	−27	21	33	1,109	−6.1955
ReHo					
Hippocampus	39	−24	−15	22	−4.6154
Middle frontal gyrus	−36	57	−9	24	−4.1374
Middle temporal gyrus	54	−30	−3	25	−4.2458
Middle frontal gyrus	−36	51	9	37	−4.401
Superior frontal gyrus, medial	12	63	18	34	−4.1269
Middle frontal gyrus	−30	24	30	22	−4.7322
Superior frontal gyrus, medial	6	27	63	35	−4.4297

#### ALFF: ASD vs. HC

Specifically, compared with HC, male individuals with autism exhibited decreased amplitude of ALFF in various brain regions, including bilateral prefrontal lobes, bilateral thalamus, bilateral middle cingulate gyrus, left anterior superior temporal gyrus, right inferior temporal gyrus cortex, and increased ALFF in bilateral somatosensory association cortex, bilateral left temporal pole cortex. Conversely, in female patients with ASD, ALFF was increased in the left medial frontal gyrus, medial temporal gyrus, inferior frontal gyrus of insular cortex, right orbital medial frontal gyrus, and left suproccipital gyrus.

#### DC: ASD vs. HC

As compared to HC, male individuals with ASD showed decreased DC in bilateral cingulate cortex, middle occipital gyrus, and dorsolateral prefrontal cortex. On the other hand, DC was increased in bilateral medial prefrontal cortex, bilateral temporal pole, superior temporal gyrus, posterior cingulate gyrus, thalamus, left suproccipital gyrus, and right superior limbic gyrus. Conversely, female individuals with ASD displayed decreased DC in bilateral inferior temporal gyrus, anterior cingulate cortex, medial cingulate cortex, and temporal pole, while DC was increased in bilateral medial prefrontal cortex, posterior cingulate cortex, right superior limbic gyrus, inferior temporal gyrus and lateral prefrontal cortex.

#### ReHo: ASD vs. HC

As compared to HC, male individuals with ASD showed reduced ReHo in bilateral medial superior frontal gyrus and right medial frontal gyrus. Conversely, female individuals with ASD exhibited decreased ReHo in dorsolateral prefrontal cortex, and increased in the bilateral medial prefrontal cortex, thalamus, precuneus cortex, occipital lobe, and right inferior temporal gyrus.

### Correlation between functional activity characteristics and clinical social performance

The results of SVR prediction model indicated that several the neuroimage features in both male and female individuals could serve as significant predictors of clinical performance scores. Moreover, the specific brain regions of ALFF recognition in males and females could be found to be better fit the clinical scores of ADI-R-ONSET (female ALFF: *p* = 8.78E-07 mean = 1.28 MSE =3.41; male ALFF: *p* = 4.67E-10 mean = 1.56 MSE =3.26), *p* is defined as the significance of the maximum explicable variance information of random permutations in 10,000 random PLS replacement tests. *p*-perm was defined as the significance of the correlation coefficient between the weight value of genes and the value of brain functional activity under the n dimension in 10,000 random displacement tests (please refer to Table 3 for details). It is worth mentioning that we also found that certain brain regions identified by ReHo were able to better distinguish between female individuals with ASD and HC (mean ACC = 93.9%, mean AUC = 0.95) (for details, please refer to Table 4).

**Table 3:** Prediction of individual clinical performance score using brain functional activity based on support vector regression.

fMRI features	Clinical index	Predicted analysis			Validation analysis		
		*p-*value	rMSE	Mean	*p-*value	rMSE	Mean
Female *ReHo*	ADI_R_RRB	0.02	2.60	5.5	0.29	2.84	5.53
	SRS_AWARENESS	0.006	4.64	12.8	0.37	6.57	12.8
	SRS_MOTIVATION	0.02	4.93	14.2	0.42	7.87	14.2
	SCQ_TOTAL	0.02	9.05	18.8	0.54	9.80	18.8
Female *DC*	ADI_R_ONSET_TOTAL	6.5e-06	1.17	3.39	0.16	1.35	3.33
	SRS_COMMUNICATION	0.002	8.80	34.1	0.34	14.9	34.1
Female ALFF	ADI_R_RRB_TOTAL	6.25E-05	2.18	5.30	0.08	2.55	5.50
	SCQ_TOTAL	0.008	7.66	18.8	0.16	8.88	19.5
Male *ReHo*	ADOS_TOTAL	0.03	4.38	12.4	0.07	4.52	12.4
Male *DC*	ADI_R_RRB_TOTAL	1.39e-09	2.50	5.71	0.39	2.70	5.67
	ADOS_SOCAFFECT	0.02	3.95	9.73	0.27	4.32	9.72
Male *ALFF*	ADI_R_RRB_TOTAL	0.01E-20	2.46	5.67	0.14	2.52	5.65
	ADI_R_ONSET_TOTAL	4.67E-10	1.25	3.26	0.18	1.30	3.23

**Table 4:** Classification accuracy based on brain functional activity.

	Female		Male	
	ACC mean	AUC mean	ACC mean	AUC mean
ReHo	93.9%	0.95	69.7%	0.69
DC	87.1%	0.84	70.0%	0.68
ALFF	81.4%	0.80	71.3%	0.70

	Validate female using male’s ROI		Validate male using female’s ROI	
	ACC mean	AUC mean	ACC mean	AUC mean
ReHo	56.5%	0.53	65.9%	0.67
DC	69.2%	0.72	59.1%	0.57
ALFF	63.8%	0.59	60.3%	0.58

### Correlation between abnormal brain functional activity and gene expression profile

The whole brain transcriptome data set provided by AHBA was used to construct the “bridge” between brain mapping and gene expression. Since only 2 out of 6 donors in AHBA provided samples from the right hemisphere of the brain, we restricted our analysis to the left hemisphere data only. After rematching the gene transcription profile using the Brodmann atlas, a brain region-gene transcription expression matrix was finally generated (the size was 40 region *13561 gene expression; gene samples were not collected in BA29 of the Brodmann atlas, therefore only the gene expression values of 40 brain regions in the left brain were utilized).

**Figure 2:**
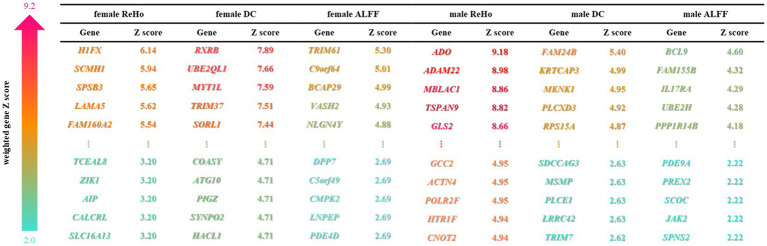
Weighted genes associated with autism. PLS recognizes the top 5% weighted genes of male and female brain functional characteristics, and sorts them according to gene weight Z-scores, and displays the top 5 and the bottom 5 genes of Z-scores. The depth of the color represents the Z-score.

**Figure 3:**
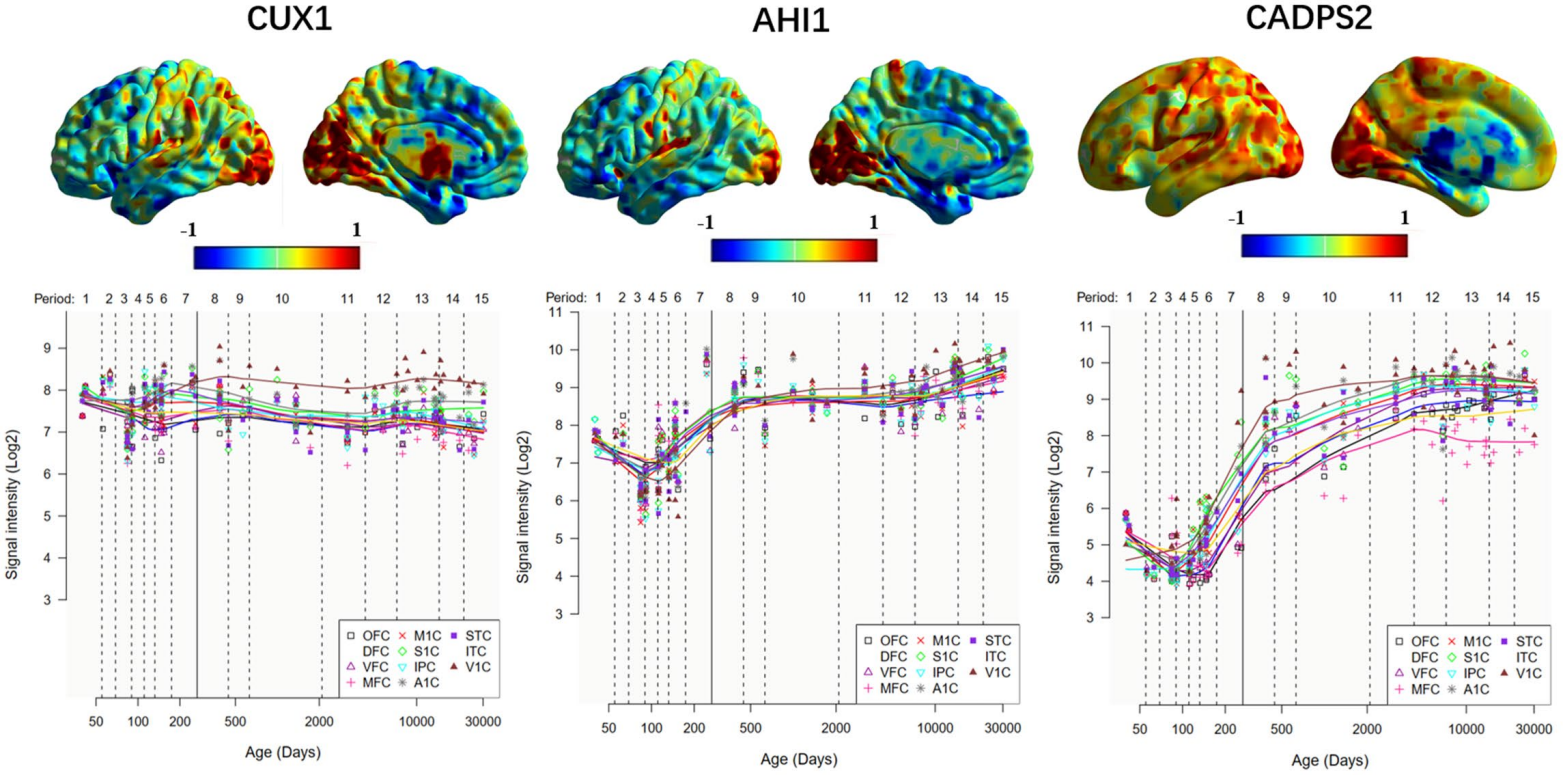
Expression of ASD risk genes in the left hemisphere and temporal -specific distribution in 11 cerebral cortices. The horizontal axis represents time (days); the vertical axis represents gene expression level, and different colored broken lines represent gene expression in different neocortical regions. OFC, orbital prefrontal cortex; DFC, dorsolateral prefrontal cortex; VFC, ventrolateral prefrontal cortex; MFC, medial prefrontal cortex; M1C, primary motor (M1) cortex; S1C, primary somatosensory (S1) cortex; IPC, posterior inferior parietal cortex; A1C, primary auditory (A1) cortex; STC, superior temporal cortex; ITC, inferior temporal cortex; V1C, primary visual (V1) cortex.

Next, PLS regression analysis was used to characterize the spatial similarity between ASD heterogeneous brain functional activity and gene expression profiles. The components with significant results after two spatial autocorrelation corrections were taken as functional activity features that could effectively explain the variance information of total gene expression. Specifically, the following components yielded noteworthy outcomes: (Male ReHo: PLS1 explains 43.7% variance, *p* = 0.003, *p*-perm = 0; Male ALFF: PLS2 explained 25.3% variance, *p* = 0.03, *p*-perm = 0.0007; Male DC: PLS2 explained 27.1% variance, *p* = 0.02, *p*-perm = 0.00005; Female ReHo: PLS2 explained 30.6% variance, *p* = 0.02, *p*-perm = 0.0001; Female ALFF: PLS4 interpretation 16.7% variance, *p* = 0.003, *p*-perm = 0.002; Female DC: PLS1 explained 36.8% variance, *p* = 0.02, *p*-perm = 0) (Figures 2, 3).

**Figure 4:**
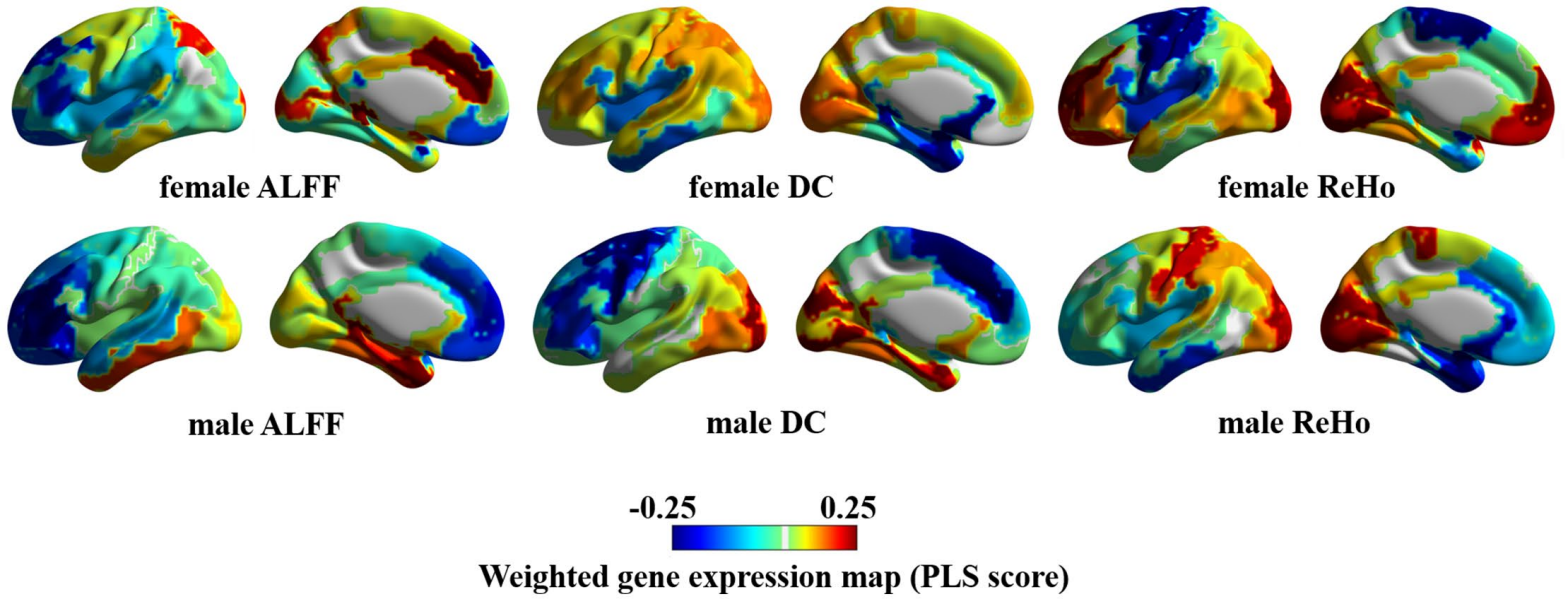
Weighted gene expression in the left hemisphere.

The similarity and personality between PLS weighted distribution of genes in male and female ASD bring another mechanism to our attention. Specifically, male individuals with ASD exhibited an evident gradient change in the whole-brain weighted gene expression map of ALFF, DC, and ReHo, whereas female individuals with ASD displayed an insignificant gradient change and even an opposite change in some regions associated with ALFF, DC, and ReHo (Figure 4).

### Enrichment analysis of ASD-related genes

**Figure 5:**
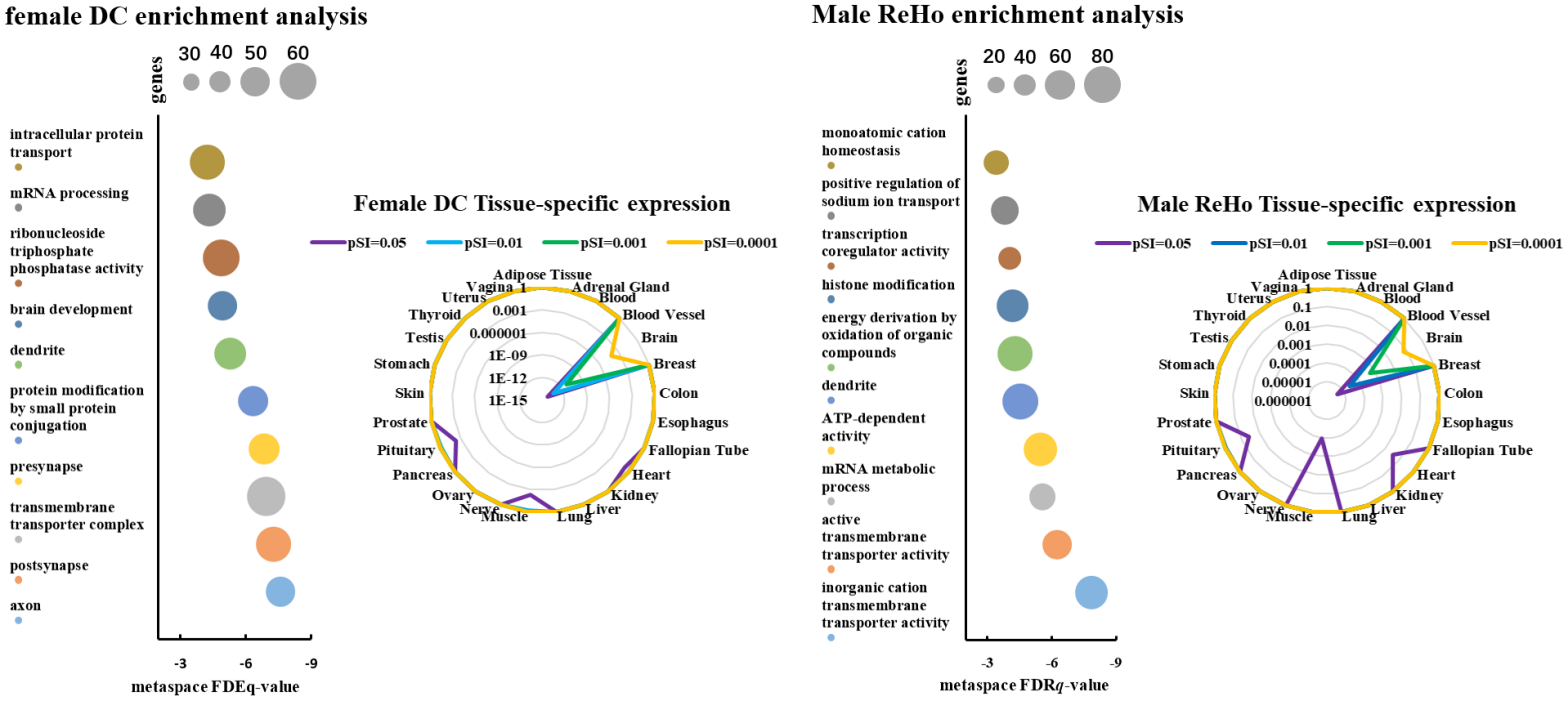
The scatter plot represents the genetic ontology term enrichment analysis of PLS, and the size of the circle represents the number of genes involved in the term. The radar map shows the specific expression levels of weighted genes in 26 tissues.

**Figure 6:**
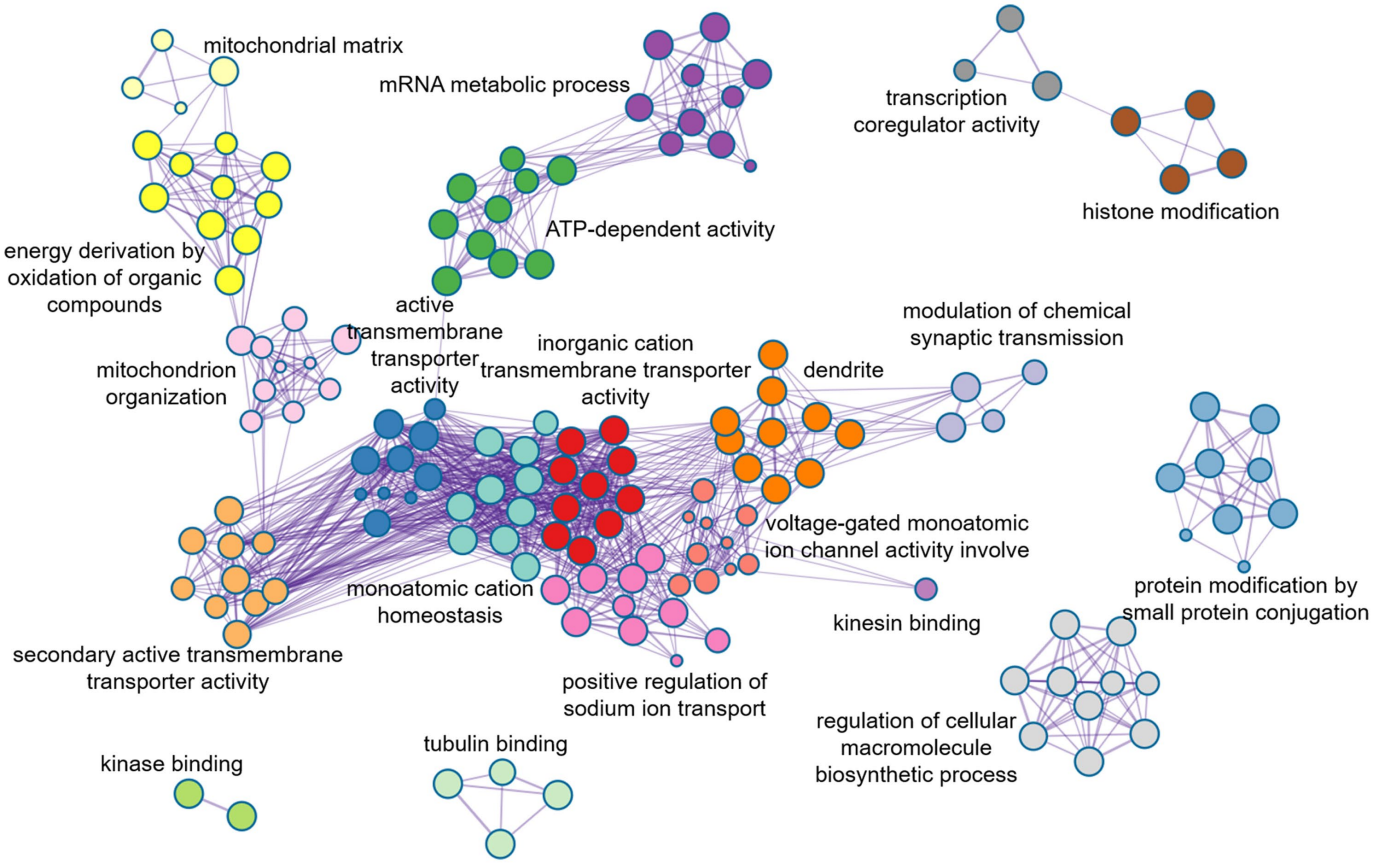
Metaspace was used to visualize the enrichment patterns of weighted genes, showing the link between molecular function and molecular mechanism in male ReHo.

**Figure 7:**
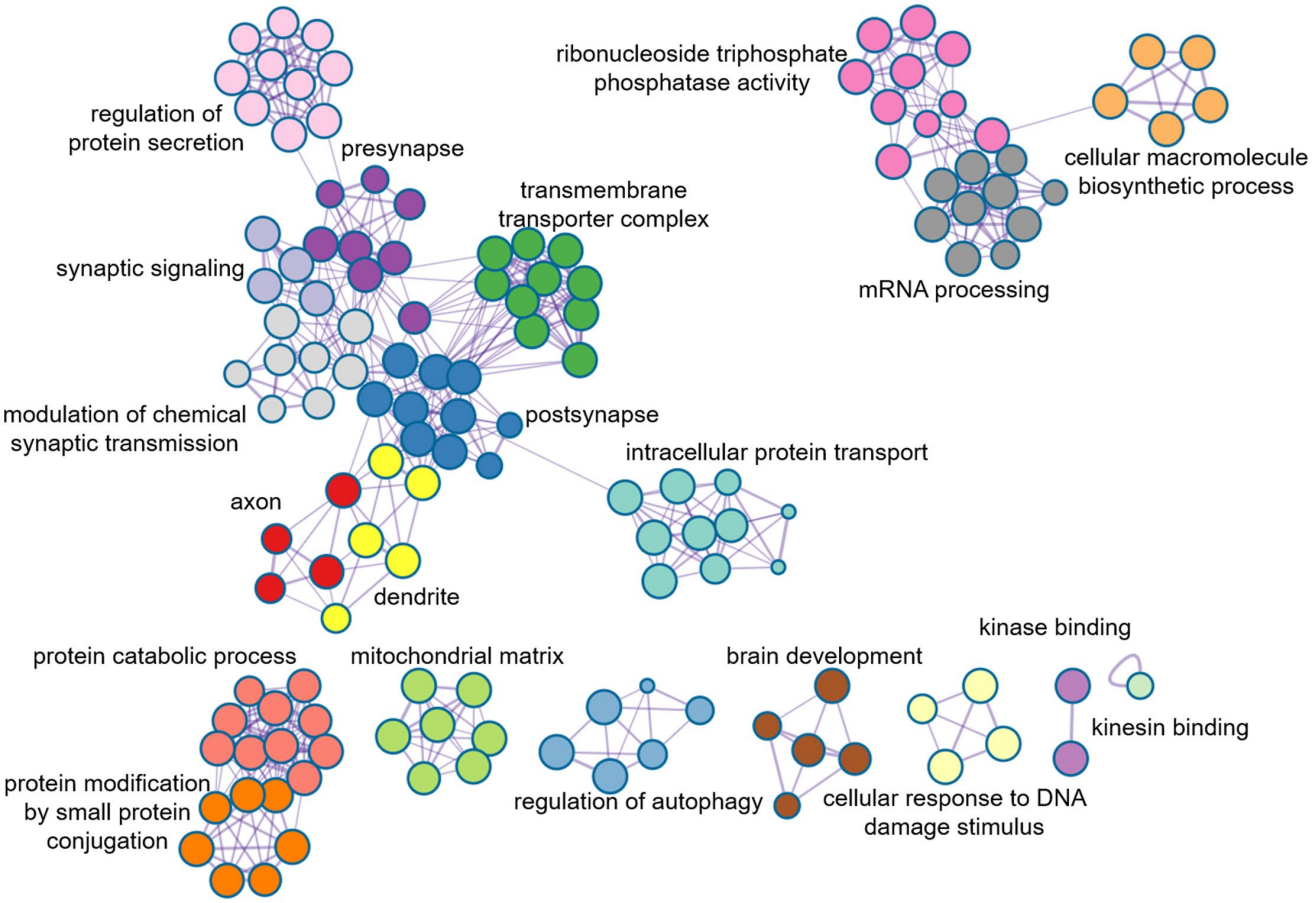
Metaspace was used to visualize the enrichment patterns of weighted genes, showing the link between molecular function and molecular mechanism in female DC.

In order to further evaluate the biological function ontology terms of genes related to ASD brain imaging characteristics, A total of 631 genes with a gene weight among the top 5% were input into the Metaspace website. We found that the genes associated with female ALFF and DC were involved in the synaptic structure and synaptic membrane of neurons, whereas those associated with male ReHo are involved in the transmembrane transporter activity of inorganic cations and other typical important processes (Figures 5–7). In addition, tissue-specific expression analysis was performed on the identified related genes in males and females, revealing that the genes associated with ASD, identified by female DC and male ReHo, exhibited a significantly specific expression pattern in the brain (Figure 5).

## Discussion

In this study, we aimed to explore alterations in neuroimaging and genetic transcription patterns in ASD patients, stratified by gender. To achieve the task, we utilized multiple-sites resting state fMRI analyses and employed ComBat to remove the “scanner effect” by a large sample data set. Through the study, we revealed multiple heterogeneous cerebral cortical regions with distinct spatial patterns of brain activity in male and female patients. Additionally, we also discovered that the functional activity of the heterogeneous brain regions identified in female patients was more effective in distinguishing ASD from HC. It is worth mentioning that the transcriptome-neuroimaging association analysis indicated that sex differences lead to both similarities and differences in gene expression patterns.

Overall, our findings suggest that atypical spontaneous brain activity in autistic patients is affected by gender, and varies in heterogeneous brain regions. Investigating the gene expression patterns in distinct brain regions may further prove to be a crucial approach in decoding the physiological origins of autism.

As is well known, brain is composed of various interconnected regions with regulatory mechanisms that form complex networks. One such network is the default mode network (DMN), which remains active even when people are not engaged in any cognitive tasks. The DMN is also involved in emotional processing, memory, emotional processing, and other cognitive tasks (Assaf et al., 2010; Lynch et al., 2013).

The rs-fMRI was used to dissect the heterogeneous changes in DMN brain regions caused by gender differences in ASD and we found that male and female patients showed heterogeneous changes in some core brain regions of the DMN. Our analysis of DMN networks in both male and female autistic patients revealed that male patients had higher low-frequency functional activity in the bilateral ventromedial prefrontal cortex than healthy controls, whereas female patients showed increased low-frequency functional activity in the right ventromedial prefrontal cortex. We observed that male and female have the similar global functional connectivity changes in the bilateral medial prefrontal cortex, the bilateral precuneus cortex, the bilateral posterior cingulate cortex, and the bilateral precuneus cortex. However, female do not show the same global functional connectivity increases in the dorsomedial prefrontal cortex as males. Based on the local similarity of functional connectivity, male and female ASD patients only exhibited similar changes in the dorsolateral prefrontal cortex, while female ASD patients have heterogeneous changes in DMN core regions, such as the bilateral ventromedial prefrontal cortex and bilateral precuneus. Overall, we found significant differences between males and females in the default mode network, particularly in the bilateral ventromedial prefrontal cortex, bilateral dorsolmedial prefrontal cortex, and bilateral precuneus. It is note that the medial ventromedial prefrontal cortex is involved in emotional regulation, social behavior, decision making, and cognitive control, and its dysfunction is considered to be a key factor leading to emotional anxiety disorder (Buhle et al., 2014; Motzkin et al., 2015). The dorsomedial prefrontal cortex shows relatively synaptic sensitivity in decision-making, reward, and pain processing in the brain (Hauser et al., 2015). The DMPFC and VMPFC are involved in different cognitive networks. The VMPFC forms a reward network with the amygdala and striatum, while the DMPFC forms a cognitive control network with the dorsal anterior cingulate cortex and posterior cingulate cortex (Cai et al., 2016; Wilson et al., 2018). Finally, the precuneus plays a crucial role in visuospatial processing, facilitate visual information processing in the brain, identifies the spatial position of objects, and is involved in the maintenance of working memory as well as the encoding and extraction of long-term memory (St Jacques et al., 2017; Schott et al., 2019).

It is worth mentioning that the functional activities of the different brain regions found in female with ASD can effectively correlate with some clinical assessment scores, such as SRS_AWARENESS, SRS_MOTIVATION, SRS_COMMUNICATION, etc. It suggests that these different brain regions may be involved in controlling behavioral cognitive functions such as consciousness, behavior, communication, etc. However, Next, the division of ASDs and HC in male using the female differences was unsatisfactory, which seems to indicate that the cerebral cortical network, consisting of the medial prefrontal cortex, middle temporal gyrus, fronto-parietal junction, thalamus, precuneus, and occipital lobe, is significantly different between male patients and female patients. Together, these regions affect the brain’s emotional regulation, memory processing, visual–spatial processing, social behavior, and cognitive control (Smith et al., 2013; Gratton et al., 2018).

Approximately one-third of the 20,000 or so genes that make up the human genome are active in the brain. They affect the development and specific functions of the brain, and ultimately our emotions, behaviors, and thoughts. The changes in brain functional activities in autism may be affected by various factors, such as external environmental changes, genetic factors (Cross-Disorder Group of the Psychiatric Genomics et al., 2013; De Rubeis et al., 2014), and molecular neuron changes. In this study, PLS method was utilized to conduct a spatial correlation analysis between gene transcriptomics and neuroimaging, and further to identify weighted genes associated with heterogeneous brain regions of ASDs. We found that the genes identified by female DC were mainly involved in the formation of neuronal synapses and the transmission of signals between synapses, while the genes identified by male ReHo were mainly involved in the controlling nerve potential signals, such as inorganic cationic transmembrane transporters, and regulating cationic transport. Additionally, the changes in neuronal potential were caused by the transmembrane transport of cations through cell membranes. Notably, the disruption of the neuronal excitation-inhibition balance mechanism is believed to be one of the important reasons for the abnormal neuronal activity in autism (Canitano and Pallagrosi, 2017; Sohal and Rubenstein, 2019). Moreover, we also found that these genes identified in male ReHo were involved in the mitochondrial matrix and influenced the secretion of adenosine triphosphate (ATP) in mitochondria. This unique mechanism may play an important role in the pathology of autism, as mitochondrial dysfunction is highly correlated with the severity of ASD symptoms. However, exactly how mitochondria are involved in autism remains unclear (Minshew et al., 1993; Rossignol and Frye, 2012).

The hierarchical structure of human cerebral cortex is closely related to its function. In this structure, different layers of neurons exhibit different patterns of gene expression, thus further demonstrating the tight link between structure and function (Maynard et al., 2021). In our study, we observed the marked difference in the whole-brain weighted genes between males and females with autism. We further noticed that there was a significant gradient change in the expression of weighted genes in the left brain of males, that is, from the frontal lobe to the occipital lobe, the weighted expression of genes shifted from low level to high level, whereas this change was not significant in females, and even reversed in parts of the cerebral cortex. It seems to prove that weighted genes are not evenly distributed in the cerebral cortex of ASD patients of different genders (Tamagawa et al., 2017).

The weighted genes we obtained were significantly associated with changes in abnormal brain regions, further identifying some ASD risk genes that are associated with changes in abnormal brain functional activities. The differences in the expression levels of different genes across different brain regions reflect the interaction or regulatory relationship between genes. Among these, CUX1 and AHI1 gene have high similarity in the whole brain gene expression level. CUX1 mutation may lead to mild intellectual disability and has familial heritability (Platzer et al., 2018). In Joubert Syndrome, the expression of AHI1 was significantly increased in the crossing axons of corticospinal tract and superior cerebellar peduncle (Ferland et al., 2004). It seems to suggest that the mutations in these two genes are common agents of certain mental illnesses. In addition, we note that CADPS2 is associated with ASD in abnormal brain function activity in both males and females, and that CADPS2 mediated neurotrophin factor release disorders contribute to increased susceptibility to autism (Sadakata et al., 2007a,b).

The expression of different genes in the development of human brain varies across different stages. Some genes are highly expressed in the critical period of the development of human organs, such as the embryonic period and the infant period, but the expression level is gradually reduced during adulthood. These difference in gene expression in different periods reflects the dynamic changes of the gene regulatory network (Franz et al., 2005; Glass et al., 2013). Since autism spectrum disorders are highly heritable and can be observed as early as childhood (Primiani et al., 2014; Christensen et al., 2016; Pierce et al., 2019), tracking the expression of specific genes at different times and studying their functions and regulation can provide better insights into the pathological mechanisms of autism spectrum disorders.

### Limitations

The current study examines the gender discrepancies in autism, without categorizing subjects according to their chronological age. Due to the absence of longitudinal developmental information, it is uncertain whether the diverse neural regions identified throughout one’s lifespan persist, thus introducing the potential for age-related effects on the results.

## Data availability statement

The original contributions presented in the study are included in the article/Supplementary material, further inquiries can be directed to the corresponding author.

## Ethics statement

The data used in this study is obtained from the open-source dataset from ABIDE. The ethical considerations pertaining to human participants in this study were meticulously examined and approved by the Institutional Review Board. Written informed consent was obtained from all patients/participants prior to their involvement in the study. For instance, The studies involving human participants were reviewed and approved by Ethical approval was obtained from the St. James’s Hospital/AMNCH (ref: 2010/09/07) and the Linn Dara CAMHS Ethics Committees (ref: 2010/12/07). Written informed consent to participate in this study was provided by the participants’ legal guardian/next of kin.

## Author contributions

YL developed the overall study objectives, designed the methodology, performed most of the experimental analysis, and wrote the manuscript. RL preprocessed experimental data and assisted in some experiments and manuscript writing. NW and JHG assisted in collating the data and interpreting the results. JJG reviewed and revised the manuscript. All authors contributed to the article and approved the submitted version.

## Funding

This work was supported by the National Natural Science Foundation of China (Nos. 61701078 and 62276049), Sichuan Province Science and Technology Support Program (Nos. 2019YJ0193, 2021YFG0126, and 2022YFS0180), the Medico-Engineering Cooperation Funds from University of Electronic Science and Technology of China (No. ZYGX2021YGLH014).

## Conflict of interest

The authors declare that the research was conducted in the absence of any commercial or financial relationships that could be construed as a potential conflict of interest.

## Publisher’s note

All claims expressed in this article are solely those of the authors and do not necessarily represent those of their affiliated organizations, or those of the publisher, the editors and the reviewers. Any product that may be evaluated in this article, or claim that may be made by its manufacturer, is not guaranteed or endorsed by the publisher.

## Footnotes

^1^ http://fcon_1000.projects.nitrc.org/indi/abide/

^2^ https://neurosynth.org/

^3^ http://human.brain-map.org

^4^ https://abagen.readthedocs.io/en/stable/

^5^ https://github.com/helab207/Alterations-in-Connectome-Dynamics-in-ASD-main

^6^ https://github.com/frantisekvasa/rotate_parcellation/tree/master/Matlab

^7^ https://human.brain-map.org/microarray/search

^8^ https://neurosynth.org/genes/

^9^ https://gene.sfari.org/database/human-gene/

^10^ https://hbatlas.org/pages/hbtd

^11^ https://metascape.org/gp/index.html#/main/step1

^12^ http://genetics.wustl.edu/jdlab/tsea/
